# Self-harm among asylum seekers in Australian onshore immigration detention: how incidence rates vary by held detention type

**DOI:** 10.1186/s12889-020-08717-2

**Published:** 2020-04-30

**Authors:** Kyli Hedrick, Gregory Armstrong, Guy Coffey, Rohan Borschmann

**Affiliations:** 1 Centre for Mental Health, Faculty of Medicine, Dentistry and Health Sciences, Melbourne School of Population and Global Health, The University of Melbourne, 207 Bouverie Street, Carlton, Victoria 3010 Australia; 2 Nossal Institute for Global Health, Melbourne School of Population and Global Health, The University of Melbourne, 333 Exhibition Street, Melbourne, 3000 Australia; 3 The Victorian Foundation for Survivors of Torture (Foundation House), 4 Gardiner Street, Brunswick, Victoria 3056 Australia; 4 Justice Health Unit, Centre for Health Equity, Faculty of Medicine, Dentistry and Health Sciences, Melbourne School of Population and Global Health, The University of Melbourne, 207 Bouverie Street, Carlton, Victoria 3010 Australia; 5 Centre for Adolescent Health, Murdoch Children’s Research Institute, 50 Flemington Road, Parkville Victoria, Melbourne, 3052 Australia; 6 Health Service and Population Research Department, Institute of Psychiatry, Psychology & Neuroscience, King’s College London, London, UK; 7 Melbourne School of Psychological Sciences, The University of Melbourne, Carlton, Victoria 3010 Australia

**Keywords:** Self-harm, Asylum seekers, Immigration detention, Australia, Mental health

## Abstract

**Background:**

Detained asylum seekers are at increased risk of self-harm, and the type of detention in which they are held may further exacerbate this risk. In Australia, there are four types of closed (or held) immigration detention for people seeking asylum, with varying levels of security and supports: Immigration Detention Centres [IDCs], Immigration Transit Accommodation [ITAs], Immigration Residential Housing [IRH], and Alternative Places of Detention [APODs]. The objective of this study was to examine the variation in the incidence and method(s) of self-harm among asylum seekers in Australian onshore immigration detention, according to the type of detention in which they are held.

**Methods:**

We obtained data on all self-harm incidents reported among asylum seekers in Australian onshore immigration detention according to held detention type, as well as individual facility, between 1 August 2014 and 31 July 2015, by Freedom of Information. We calculated self-harm episode rates per 1000 asylum seekers using the average population figures for held detention type, as well as for each individual facility comprising the main types of held detention. Method(s) used to self-harm was also extracted for the main sub-populations.

**Results:**

The study included 560 episodes of self-harm. Individual facility rates of self-harm ranged from 91 per 1000 asylum seekers (95% CI 72–110) in Yongah Hill IDC to 533 per 1000 asylum seekers (95% CI 487–578) in Perth IDC. On average, calculated self-harm episode rates were highest among asylum seekers in: Immigration Transit Accommodation facilities, 452/1000 (95% CI 410–493); Alternative Places of Detention, 265/1000 (95% CI 233–296); and Immigration Detention Centres, 225/1000 (95% CI 195–254). The most frequently reported methods of self-harm across the main types of held detention were: cutting (35.2%), self-battery (34.8%), and attempted hanging (11.1%).

**Conclusions:**

Self-harm rates for asylum seekers in all types of closed immigration detention were many times higher than rates found in the general population. Average rates were not lower in facilities with lower security features.

## Background

In response to recent surges in migration, many countries around the world apply various forms of administrative detention to asylum seekers who arrive on their territories [1]. In Australia, mandatory immigration detention - both onshore and offshore, as dictated by government policy - has been applied to all ‘unlawful non-citizens’ for over 25 years [2]. During periods where there were a large number of asylum seekers arriving by boat, the vast majority of the detained population were asylum seekers [1]. Australia’s offshore immigration detention facilities housed on the Pacific island of Nauru and Manus Island, in Papua New Guinea, have attracted a great deal of attention [3], particularly regarding detention conditions and remoteness of location, and the impact these factors may have on asylum seekers’ physical and mental health. Immigration detention environments in Australia are also diverse - until recently there were 15 operational individual detention facilities in the onshore detention network (subsequent to the period examined in this study, a number of facilities have closed). Each facility may present its own challenges for asylum seekers.

There are four main types of held immigration detention (comprised of the aforementioned individual detention facilities) in the Australian onshore detention network (which includes Christmas Island): (a) Immigration Detention Centres (IDCs); (b) Immigration Transit Accommodation (ITAs); (c) Immigration Residential Housing (IRH), and; (d) Alternative Places of Detention (APODs) [4]. IDCs have the highest security arrangements in the Australian onshore detention network, with high security fencing, guards, and additional forms of surveillance [4]. IDCs are used to detain asylum seekers, individuals who overstay their visas, and individuals who have had their visas cancelled or refused for character reasons [4]. ITAs are closed detention centres that may have less stringent security measures than IDCs [4]. They were initially designed to house individuals who were being transferred to other detention facilities, or who were being removed (otherwise known as being deported) [4]. IRH is classified as a secure form of detention, with lower security features than IDCs [4]. IRH provides accommodation that can cater to families, although they may also be used to house single adults. APODs are places designated by the (then-called) Department of Immigration and Border Protection [DIBP] to be used for detaining asylum seekers who are assessed as posing minimal risk to the Australian community [4]. APODS are less secure than other types of held detention, and often have a more domestic environment (generally for housing children and families) to provide individuals with more privacy and the opportunity to do things together, such as cook and eat [5].

It is now well established that immigration detention has a deleterious impact on the psychological well-being of many asylum seekers [6–8], and that these adverse effects may be enduring [9]. Until recently, however, little research regarding self-harm in the Australian asylum seeker population existed. A 2017 study of self-harm among asylum seekers in onshore immigration detention reported a self-harm episode rate of 224 per 1000 detained asylum seekers in the 20-month period to May 2011 [10]. Our more recent research into self-harm across the whole Australian asylum seeker population between 1 August 2014 and 31 July 2015 [11] reported a self-harm episode rate of 257 per 1000 detained asylum seekers in onshore immigration detention, representing a 15% increase in self-harm rates since May 2011. This self-harm episode rate was also calculated to be 214 times the Australian community rate for hospital-treated self-harm during the same period [11]. For further comparison, our calculated self-harm episode rates among community-based asylum seekers (5 episodes/1000 asylum seekers), and those held in community detention (27 episodes/1000 asylum seekers) were 4 and 22 times the Australian community rates for hospital-treated self-harm, respectively [11]. Such findings are consistent with research highlighting that asylum seekers are at increased risk of self-harm [12], and that being detained further exacerbates this risk [13, 14].

The findings from our earlier study convey important information about the elevated risk of self-harm among asylum seekers in the onshore immigration detention population as a whole, in comparison with both asylum seekers in community-based arrangements and community detention [11], as well as the general Australian population. They do not, however, provide any insight into the influence different types of held detention might have on the incidence of self-harm among asylum seekers. Nor do they permit scrutiny of self-harm rates among asylum seekers at the individual detention facility level. In this way, the unique influence that different types of held detention might have on rates of self-harm have thus far been obscured, by treating the entire onshore detention network as one homogenous environment. As such information may assist in the prevention of self-harm among detained asylum seekers, it warrants urgent attention. Indeed, research from the prison literature has found that detention type or setting impacts on self-harm risk among incarcerated adults [14]. Specifically, self-harm risk has been found to be associated with higher security detention facilities (particularly for men) [14], transit or remand centres [14, 15] (where transfers to or from settings may occur more frequently), and mixed detention facilities (particularly for women) [14]. Asylum seekers in the Australian onshore detention population are housed in both high and low security centres, as well as in different types of transit and mixed accommodation; these detention environments may differentially impact on self-harm risk. The aim of the current study was therefore to build and extend upon our earlier research into self-harm in the onshore immigration detention population as a whole, by examining the variance in the incidence and method(s) of self-harm among asylum seekers in onshore immigration detention according to held detention type, and each individual facility comprising each type. Whilst the vast majority of ‘unlawful non-citizens’ detained in onshore immigration detention during the study period were asylum seekers, referred to as ‘irregular maritime arrivals’ (IMAs) (accounting for at least 70% of the entire population – the average annual composition of the population was 70% IMAs, 2.4% air arrivals, 12.2% visa cancellations, 17.3% overstayers, 0.1% seaport arrivals, and 0.02% illegal foreign fishers [16]) - there was a remaining group who were detained for various other reasons, some of whom were also asylum seekers (e.g., some visa cancellations will also have been asylum seekers). Given the composition of onshore immigration detention during this time, and for the sake of brevity, we will refer to the overall population in this study as asylum seekers in Australian onshore immigration detention.

## Methods

We obtained data on all self-harm incidents reported among asylum seekers in Australian onshore immigration detention between 1 August 2014 and 31 July 2015, by Freedom of Information [FOI] [17]. These data were published on the Department of Immigration’s FOI disclosure log [18]. Ethics approval for this study was obtained from the University of Melbourne’s Human Research Ethics Committee (#1749949.1).

We undertook a content analysis study of all self-harm incidents, with self-harm defined as all forms of intentional self-injury (or self-poisoning), irrespective of suicidal intent or motivation [11, 19]. Parts of this methodology have been described extensively elsewhere (see [11]). For this study, information regarding held detention type, and individual facility were extracted from the details provided in each incident report. Whilst community detention is classified as a type of held immigration detention [4], it was not included in this study as we have previously examined the incidence of self-harm in this population elsewhere [11]. Details relating to age and country of origin were not able to be extracted from the incident reports. This potentially identifying information, if noted, would have been redacted prior to the incidents being released via Freedom of Information. Details regarding country of origin relating to the whole onshore immigration detention population were instead sourced from official DIBP [16] statistics. As the Department of Immigration does not publish country of origin information for each type of detention, or at the individual facility level, we were not able to report such details. The incident reports available for analysis did not include suicide. (The most definitive source of deaths among Australian asylum seekers, the Australian Border Deaths Database [20], reports that there were three suicides by asylum seekers during the study period. All three deaths occurred among asylum seekers living in the Australian community). One self-harm episode was reported as occurring in Curtin IDC, however as the official population figures [16] for this facility were recorded as zero for the entire 12 month period (with those held at the detention centre transferred to other facilities at the beginning of the study period, and the centre closing sometime after this), this incident was not included in the analysis. Ten percent of the incident reports were double-coded by an independent coder. Inter-rater reliability was established to be high (kappa = 0.95), and based on this, all remaining incidents were coded by a single coder (KH) [21].

We calculated the episode rate of self-harm per 1000 detained asylum seekers for each held detention type, using the average adult population figures [16] (with 95% Confidence Intervals [CI]). Self-harm episode rates were also calculated for each individual facility comprising each held detention type, where population figures were provided by the DIBP. Numbers were too low to calculate episode rates for IRHs.

## Results

### Demographics

Between 1 August 2014 and 31 July 2015, there were a total of 560 self-harm episodes among asylum seekers in Australian onshore immigration detention (231 episodes (41.2%) by males, 90 (16.0%) by females, and 239 episodes (42.6%) where gender could not be determined) (see Table 1). According to official statistics [16], the two major source countries of asylum seekers detained in Australian onshore immigration detention were Iran and Sri Lanka. One in five (19.6%) asylum seekers held in onshore detention had been detained for greater than 2 years.

**Table 1:** The number of self-harm episodes among asylum seekers in Australian onshore immigration detention between 1 August 2014 and 31 July 2015 by held detention type, individual detention facility and gender

	Number (%)			
	Males (*n* = 231)	Females (*n* = 90)	Gender not specified (*n* = 239)	Total (*N* = 560)
Wickham Point IDC	11 (4.7)	1 (1.1)	15 (6.3)	27 (4.8)
Maribyrnong IDC	24 (10.3)	19 (21.1)	18 (7.7)	61 (10.8)
Perth IDC	8 (3.4)	–	8 (3.3)	16 (2.8)
Christmas Is IDC	50 (21.6)	–	46 (19.2)	96 (17.1)
Villawood IDC	37 (16.0)	11 (12.2)	15 (6.2)	63 (11.2)
Yongah Hill IDC	25 (11.0)	–	12 (5.0)	37 (7.0)
**IDCs Total**	**155 (67.0)**	**31 (34.4)**	**114 (47.7)**	**300 (53.7)**
Adelaide ITA	**–**	**–**	4 (1.7)	4 (0.7)
Brisbane ITA	10 (4.5)	2 (2.2)	17 (7.1)	29 (5.2)
Melbourne ITA	14 (6.0)	21 (23.3)	30 (12.6)	65 (11.6)
**ITAs Total**	**24 (10.5)**	**23 (25.5)**	**51 (21.4)**	**98 (17.5)**
**Sydney IRH (Total)**	**1 (0.5)**	**1 (1.1)**	**1 (0.4)**	**3 (0.5)**
Wickham Pt APOD	38 (16.4)	16 (17.7)	40 (16.8)	94 (16.7)
Blaydin APOD	5 (2.1)	8 (9.0)	4 (1.7)	17 (3.0)
Inverbrackie APOD	1 (0.5)	4 (4.4)	9 (3.7)	14 (2.5)
Phosphate Hill APOD	3 (1.2)	1 (1.1)	8 (3.3)	12 (2.1)
Construction Camp APOD	4 (1.8)	6 (6.6)	12 (5.0)	22 (4.0)
**APODs Total**	**51 (22.0)**	**35 (39.0)**	**73 (30.5)**	**159 (28.3)**

### Episode rates of self-harm by held detention type and individual detention facility

Self-harm episode rates per 1000 asylum seekers (and corresponding 95% confidence intervals [CIs]) according to held detention type were ITAs: 452 episodes/1000 asylum seekers (95% CI 410–493); APODs (combined mainland and Christmas Island total): 265 episodes/1000 asylum seekers (95% CI 233–296); and IDCs: 225 episodes/1000 asylum seekers (95% CI 195–254). In individual facilities, the episode rate was highest in: Perth IDC, 533 episodes/1000 asylum seekers (95% CI 487–578); and lowest in Yongah Hill, 91 episodes/1000 asylum seekers (5% CI 72–110) (see Table 2).

**Table 2:** Self-harm episode rates among asylum seekers in Australian onshore immigration detention between 1 August 2014 and 31 July 2015, as well as the average adult population figures, by held detention type and individual detention facility

	Average adult population figures*	Episode rate per 1000	CI (95%)
Wickham Point IDC**	93	290	256–323
Maribyrnong IDC	119	512	467–556
Perth IDC	30	533	487–578
Christmas Island IDC	331	290	256–323
Villawood IDC	354	178	152–204
Yongah Hill IDC	406	91	72–110
**IDCs Total**	**1333**	**225**	**195–254**
Adelaide ITA	23	173	147–198
Brisbane ITA	62	467	425–509
Melbourne ITA	132	492	447–536
**ITAs Total**	**217**	**452**	**410–493**
APODs Total***	**601**	**265**	**233–296**

*The DIBP [19] statistics these population figures are extracted from refer to asylum seekers as “illegal maritime arrivals”. Total average population figures for the onshore immigration detention population displayed here vary slightly (by 1%) to those we have reported elsewhere [11] due to a lag in the DIBP’s reporting of figures for individual facilities and/or held detention types.** Wickham Point IDC did not record population figures from January 2015 onwards, so calculated episode rates are for a 5 month period only. *** A combined self-harm episode rate was calculated for APODs on the mainland, as well as for APODs on Christmas Island. Whilst APODs on Christmas Island were reported to have closed in late 2014, self-harm incidents were recorded as occurring in these facilities after this time

### Methods of self-harm by held detention type

Information regarding method(s) used to self-harm was extracted from 468 (83.5%) of the 560 episodes of self-harm among asylum seekers in the Australian onshore immigration detention population. Of the nine different types of methods identified, the three most frequently reported were: cutting (35.2%), self-battery (34.8%), and attempted hanging (11.1%) (Table 3).

**Table 3:** Methods of self-harm used by asylum seekers in Australian onshore immigration detention between 1 August 2014 and 31 July 2015, by held detention type

	Number (%)			
	Immigration Detention Centres (IDCs) (*n* = 270)	Alternative Places of Detention (APODs) (*n* = 123)	Immigration Transit Accommodation (ITAs) (*n* = 75)	Total (*n* = 468)
Cutting	109 (40.3)	31 (25.2)	25 (33.3)	165 (35.2)
Self-battery	92 (34.0)	45 (36.5)	26 (34.6)	163 (34.8)
Hanging	30 (11.1)	13 (10.5)	9 (12.0)	52 (11.1)
Self-poisoning (medication)	16 (6.0)	18 (14.6)	4 (5.3)	38 (8.1)
Self-poisoning (chemicals)	8 (3.0)	10 (8.1)	7 (9.3)	25 (5.3)
Ingesting foreign object	6 (2.2)	2 (1.6)	–	8 (2.0)
Burning	6 (2.2)	–	3 (4.0)	9 (1.9)
Lip sewing	3 (1.1)	–	–	3 (0.6)
Jumping from height	–	4 (3.2)	1 (1.3)	5 (1.0)

### Site of injury

The site of bodily injury was determined in 346 (74.0%) of the 468 self-harm episodes among asylum seekers where method was known. The most common site of injury across all held detention types in Australian onshore immigration detention was the head (143 episodes, 41.3%), followed by the wrist and arm (103 episodes, 30.0%), and the neck (50 episodes, 14.3%) (see Table 4).

**Table 4:** Site of bodily injury in self-harm episodes among asylum seekers in Australian onshore immigration detention between 1 August 2014 and 31 July 2015, by held detention type

	Number (%)			
	Immigration Detention Centres (IDCs) (*n* = 209)	Alternative Places of Detention (APODs) (*n* = 83)	Immigration Transit Accommodation (ITAs) (*n* = 54)	Total (*n* = 346)
Head	77 (37.0)	44 (53.0)	22 (40.7)	143 (41.3)
Wrist and arm	69 (33.0)	18 (21.8)	16 (29.6)	103 (30.0)
Neck	31 (14.8)	12 (14.4)	7 (12.9)	50 (14.3)
Hand and fingers	24 (11.4)	7 (8.4)	4 (7.4)	35 (10.0)
Chest and torso	2 (1.0)	–	2 (3.7)	4 (1.1)
Stomach	3 (1.4)	2 (2.4)	–	5 (1.4)
Leg	3 (1.4)	–	2 (3.7)	5 (1.4)
Foot	–	–	1 (2.0)	1 (0.5)

## Discussion

To our knowledge, this is the first examination of the variation in the incidence and method(s) of self-harm among asylum seekers in the Australian onshore immigration detention population by held detention type, as well as for each individual facility comprising each type. Between 1 August 2014 and 31 July 2015, there were 560 episodes of self-harm among asylum seekers in Australian onshore immigration detention**.** Calculated individual facility rates ranged from 91 episodes/1000 detained asylum seekers (Yongah Hill IDC) to 533 episodes/1000 asylum seekers (Perth IDC). On average, self-harm episode rates were highest among asylum seekers in ITAs (452 episodes/1000 asylum seekers), followed by APODs (265 episodes/1000 asylum seekers), and IDCs (225 episodes/1000 asylum seekers). Across all three types of held detention, cutting, self-battery, and attempted hanging were the three most frequently reported methods used to self-harm.

### Rates of self-harm

The episode rates of self-harm we have calculated for all three types of held immigration detention are considerably higher than the episode rates of hospital-treated self-harm – 1.2 per 1000 – in the general Australian community during a similar period [22]. Calculated self-harm episode rates among asylum seekers detained in IDCs, APODs and ITAs are therefore 187 times, 220 times and 376 times the hospital-treated rates of self-harm in the general Australian community, respectively. These findings are consistent with previous research [6, 12], including our own [10, 11], which highlights that detained asylum seekers are at an elevated risk of self-harm in comparison to the general community. The findings of the present study also extend upon earlier research, by highlighting that rates of self-harm in asylum seekers across all three types of held onshore immigration detention in Australia are exceptionally high in comparison with rates found in the general community.

### Rates of self-harm in the three main types of held detention

Whilst self-harm episode rates for asylum seekers across all three main types of held immigration detention were markedly higher than hospital-treated rates of self-harm in the general Australian population, interestingly, average self-harm rates were not lower in types of detention with lower security arrangements. Indeed, it might be expected that lower security facilities, with less stringent security features, and more flexible accommodation, for families or other vulnerable individuals, might be associated with a lowered risk of self-harm in asylum seekers. However, this was not the pattern observed in the present study: on average, self-harm episode rates were highest in ITAs (452 episodes/1000 asylum seekers), followed by APODs (265 episodes/1000 asylum seekers), the two types of detention with lower security, and purportedly more flexible accommodation arrangements. These findings expand upon earlier understandings of self-harm risk among detained asylum seekers [6, 12], including our own research [10, 11], to highlight that types of detention may differentially impact on self-harm risk among asylum seekers. What these findings speak to, consistent with research from the prison literature [14, 15], is that whilst detention increases the overall risk of self-harm, particular features of detention environments are associated with such risk. Drawing out the particular features of the detention arrangements that may have influenced the rates of self-harm found in the present study may help guide future self-harm prevention strategies among asylum seekers, and are therefore important to highlight.

### Rates of self-harm among asylum seekers held in ITAs

Whilst ITAs were originally designed as low security short-term detention facilities for people who arrived by air, but who were not immigration cleared [23], they were subsequently expanded to include asylum seekers who arrived by boat, including children [24]. Individuals may be held in ITAs whilst in transit (for example, whilst awaiting medical treatment - including those transferred from Nauru or Manus Island - or accompanying a family member who is receiving treatment, prior to being transferred to another onshore or offshore facility), or on a short-term or long-term basis, whilst awaiting the processing of an asylum claim [24]. Research from the prison literature [14] has observed that rates of self-harm among incarcerated adults are highest among those who are detained in environments that contain mixed populations (for example, facility types housing both men and women, with large age ranges, and including remand and sentenced detainees), or when length or duration is uncertain (such as transit or remand accommodation) [15]. As the ITAs contain very mixed populations, housed for short, medium and long-term periods, it is plausible that these features of the detention environment may have contributed to the high rates of self-harm found in these types of detention. As the prison literature also indicates that rates of self-harm maybe higher in more secure facilities (particularly for men) [14], however, our findings are only partially consistent with such research. The underlying gender composition of those detained in ITAs during the study period may have influenced the pattern of findings observed in our study. Further research is needed to better understand self-harm risk, including the gendered dimensions of such risk, by held detention type.

Prior research has also documented that the practice of transferring asylum seekers between or within facilities, without adequate time to inform family, friends or legal representatives, often resulting in the separation of family members, causes significant distress [5], and may precipitate episodes of self-harm [10]. Given these same conditions and practices are found in ITAs, it is likely that these may also have influenced the high rates of self-harm identified in the present study among asylum seekers held in ITAs. Whilst the practice of transferring asylum seekers between or within facilities occurs across the immigration detention network, the mix of individuals in the populations at ITAs, as well as the circumstances of their internment, is more diverse than in other detention types.

In short, there are a range of distinct features associated with ITAs – diverse, mixed populations, detained for variable amounts of time, and for a range of reasons, and with frequent separations from family for unknown amounts of time. As these are features of detention environments that have previously been established to cause distress, and precipitate self-harm in detained populations, they are likely to also have influenced the high rates of self-harm found among those detained in ITAs in the current study. It is also conceivable that the lower levels of security in such facilities also provided individuals with greater opportunity to self-harm (easier access to means, less observation or possibility of being detected, for example).

### Rates of self-harm among asylum seekers held in APODs

APODs are places designated by the DIBP to be used for detaining asylum seekers that are less secure than other types of held detention, and purportedly have a more domestic environment (generally for housing children and families), to allow more privacy, and the opportunity to do things together [4]. Reports [5, 25] from the period in question indicate, however, that APODs were often immigration detention centres that were given a number of superficial improvements (for example, being cleaned and made more functional) and accompanied by a name change. Testimonies from mental health clinicians who have worked in APODs have also highlighted that whilst APODs were meant to provide a more supportive environment for the most vulnerable asylum seekers (including children and families), these closed, institutionalised environments were not conducive to managing the health of those held there [25].

Furthermore, as canvassed at length in the 2014 Australian Human Rights Commission [AHRC] National Inquiry into Children in Immigration Detention [5], closed detention environments, such as APODs, often result in parental disempowerment, as parents are not allowed control or authority, even over the most routine of everyday matters, such as accessing nappies, bottles and baby food, to other matters, such as decisions regarding schooling. As reported by the AHRC [5], for example, children detained in Christmas Island (APODs) had almost no school education between July 2013 to July 2014, something parents were powerless to alter. Indeed, the observations of those who have worked in APODs [25] have further highlighted that ‘this lack of autonomy frequently became distressing for parents’ … with ‘children quickly becoming aware of this distress, which in turn created what could be described as self-perpetuating distress and helplessness about parenting, roles and attachment’ (p. 526). Additional features of APODs observed to impact on the mental health of those detained there include serious concerns around child protection, including notifications and inadequate responses to reports of child abuse [5, 25].

The use of hotels, motels, and other temporary forms of accommodation as places of long-term detention for adults, as well as families with children, without adequate access to family, legal and social supports or mental or physical health treatment is another characteristic of APODs known to detrimentally impact on the mental health of those detained under such arrangements [26]. The practice of using hotels as places of long-term detention with extremely restrictive conditions, particularly for asylum seekers transferred to Australia from Nauru and Manus Island for medical treatment, has increased in recent years; the mental and physical health consequences of such detention arrangements are deeply concerning and warrant urgent investigation [26]. In short, it is plausible that the features associated with APODS that were found to impact on the mental health of those detained there, as outlined above, may have also influenced the high rates of self-harm found in these particular types of detention.

### Rates of self-harm among asylum seekers held in IDCs

Whilst average self-harm episode rates in IDCs (225 episodes/1000 asylum seekers) were lower than rates identified among asylum seekers in ITAs and APODs, both the lowest and highest self-harm episode rates were identified as occurring in IDC facilities, highlighting a large intra type range. The lower overall self-harm episode rates identified among asylum seekers in IDCs in the present study, compared with rates found in asylum seekers in ITAs, could relate to the fact that closed, secure environments, with more surveillance and staffing levels, mean that self-harm can be more closely monitored and controlled. More specifically, the stricter monitoring and surveillance of asylum seekers in IDCs may have provided fewer opportunities for self-harm attempts or threats to go unnoticed by staff. In addition to this, repeat occurrences of self-harm may be more easily ‘prevented’ in these more secure facilities, by using self-harm management practices, where security guards are placed ‘at arms-length’ of an asylum seeker for long periods following an episode of self-harm, to prevent another episode from occurring [27]. Such practices are commonly used in IDCs, and have been characterised as a form of risk management from a ‘security’ perspective, rather than a clinical perspective [27]. In short, it is conceivable that the lower overall episode rates of self-harm found in IDCs, compared with those found in ITAs, could relate to the design features of IDC facilities, the higher levels of monitoring and control, as well as the types of risk management strategies used more frequently in IDCs than in other types of held detention.

#### Rates of self-harm by individual facilities

Individual facility self-harm episode rates ranged from 91 per 1000 detained asylum seekers in Yongah Hill IDC to 533 per 1000 in Perth IDC, highlighting both a large inter- and intra-facility range. Perth IDC – the individual facility with the highest self-harm rates – is primarily used to house asylum seekers who were transferred to Perth for medical treatment [28]. This includes transfers from Christmas Island (2600 kms from Perth), Curtin IDC, as well as from Nauru and Manus Island, which highlights the large distance asylum seekers need to be transported if they require important medical treatment. It is plausible, therefore, that the high episodes rates of self-harm identified among asylum seekers in the Perth IDC in the present study may have been associated with the specific conditions of this facility, including its remoteness of location (from other facilities), and the need to be transported from other facilities to be housed there – all factors previously found to cause mental distress [5], and precipitate self-harm episodes in detained asylum seekers in Australia [10]. As the population figures in Perth IDC were very low, however, it is possible that a few frequently self-harming individuals could have increased the self-harm rate identified in this facility. The high rates identified may also relate to the fact that individuals who were mentally unwell were transferred to Perth IDC from Christmas Island, Nauru or Manus Island, in order to receive psychiatric assessment on the mainland. This means that parts of the asylum seeker population in Perth IDC may have been a selected group of particularly unwell people.

It is important to note that the lowest individual facility self-harm episode rate identified in the present study among asylum seekers held in Yongah Hill IDC – 91 per 1000 detained asylum seekers – was calculated as 75 times the Australian community rate of hospital treated self-harm during the same period [22]. These findings very sharply illuminate the difference between the health of asylum seekers in Australian immigration detention facilities, using self-harm as a measure of health, and the health of Australians in the general community.

#### Methods of self-harm

The three most frequently reported methods of self-harm across the three main types of closed immigration detention were cutting, self-battery and hanging. Similarly, the three most common sites of bodily injury were consistent across the three types of closed detention: head, wrist/arm, and neck. The current findings are consistent with previous research which has documented high rates of hanging among asylum seekers detained in onshore immigration detention [10], as well as our recent research which identified high rates of hanging among both male and female asylum seekers in the Australian onshore detention population [11]. What the current findings allow us to further discern is that approximately 1 in 10 self-harm incidents in each of the three types of closed immigration detention in Australia involved hanging. As research has established that hanging is strongly associated with an elevated risk of suicide [29], the high rates of hanging – observed across all three types of closed onshore immigration detention in the current study – clearly has serious implications for the safety of asylum seekers across the entire Australian onshore immigration detention population.

#### Strengths and limitations

Our study had a number of important strengths. Firstly, these are the first published data examining self-harm among asylum seekers in Australian onshore immigration detention, by held detention type, as well as individual facility. Secondly, we had access – under Freedom of Information laws – to all self-harm incidents from across the entire Australian onshore detention population, as reported to, and archived by, the (then-called) DIBP. Thirdly, our sample was large, and permitted us to examine not only the incidence of self-harm by detention type, and individual facility, but also the characteristics of self-harm, including method(s) used to self-harm, as well as the site of bodily injury – the first published data of this kind.

Our study also had several limitations. The number of unrecorded self-harm episodes among asylum seekers in Australian onshore immigration detention is unknown. It is likely, therefore, that the rates reported here understate the incidence of self-harm among asylum seekers detained in Australia. Details regarding age or country of origin, including by each detention type, and at the individual facility level, were not able to be ascertained from the incident reports. Furthermore, gender was only able to be determined in just over half of all cases. This meant that we were not able to reliably calculate rates of self-harm by held detention type or individual facility, according to gender. Lastly, as the population figures in Perth IDC were very low, it is possible that a few frequently self-harming individuals could have increased the self-harm rate identified in this facility.

## Conclusions

Our findings illustrate the far-reaching detrimental impact that Australian onshore immigration detention has on the mental health of asylum seekers, specifically in relation to self-harm. They also highlight the particular influence that different types of held immigration detention, as well as individual facilities – and their associated features, practices and policies – may have on the health of detained asylum seekers. In conclusion, the independent surveillance and monitoring of self-harm among asylum seekers detained in Australian immigration detention – the first step in any national self-harm prevention strategy [30] – must be established in order to strengthen the data that can be used to address this major public health concern.

## Data Availability

The dataset that supports the current findings was generated by data accessed via the DIBP’s Freedom of Information Disclosure log (2017) on 3 April 2017, located at: https://www.homeaffairs.gov.au/access-and-accountability/freedom-of-information/disclosure-logs.
